# Sample size re-estimation without un-blinding for time-to-event outcomes in oncology clinical trials

**DOI:** 10.7555/JBR.31.20160111

**Published:** 2018-01-26

**Authors:** Li-Hong Huang, Jian-Ling Bai, Hao Yu, Feng Chen

**Affiliations:** 1. Department of Biostatistics, School of Public Health, Nanjing Medical University, Nanjing, Jiangsu 211166, China; 2. Ministry of Education Key Laboratory for Modern Toxicology, School of Public Health, Nanjing Medical University, Nanjing, Jiangsu 211166, China.

**Keywords:** oncology study, clinical trial, sample size re-estimation, expectation-maximization algorithm

## Abstract

Sample size re-estimation is essential in oncology studies. However, the use of blinded sample size reassessment for survival data has been rarely reported. Based on the density function of the exponential distribution, an expectation-maximization (EM) algorithm of the hazard ratio was derived, and several simulation studies were used to verify its applications. The method had obvious variation in the hazard ratio estimates and overestimation for the relatively small hazard ratios. Our studies showed that the stability of the EM estimation results directly correlated with the sample size, the convergence of the EM algorithm was impacted by the initial values, and a balanced design produced the best estimates. No reliable blinded sample size re-estimation inference can be made in our studies, but the results provide useful information to steer the practitioners in this field from repeating the same endeavor.

## Introduction

Given the life-threatening nature and the unmet medical needs of many types of cancer, the drug development process in the field of oncology should be accelerated. To this end, many clinical trial approaches have been proposed. The sample size required for a clinical trial should be sufficiently large to provide a reliable answer to the questions addressed^[1]^, and the method by which the sample size is calculated should be provided in the protocol. This method is the most basic requirement for planning all studies because it is critical to the success of a study and pertains to budget considerations. For fixed sample size designs, there is a risk that expected trial outcomes may not obtain adequate power because some uncertainty is usually associated with the parameters in the planning phase. This deficit can be remedied by re-estimating the parameters during the interim analysis and modifying the initially planned sample size if necessary^[2– 3]^.

Although various methods have been proposed and used for sample size, a basic debate remains: Should the interim data be examined with the treatment group in a blinded or un-blinded manner? Regulatory authorities certainly favor blinded sample size reassessment (BSSR) (CPMP Working Party on Efficacy of Medicinal Products^[4]^; ICH-E9 Expert Working Group^[1]^; European Medicines Agency/Committee for Medicinal Products for Human Use^[5]^) because it better preserves study integrity. Fortunately, the work of Gould and Shih^[6]^ has shown that un-blinding is not necessary to efficiently estimate within-group variance.

Nevertheless, previous studies of sample size re-estimation addressed various statistical and practical aspects of this approach, such as BSSR in non-inferiority and equivalence trials with normally distributed outcome variables and hypotheses formulated in terms of the ratio and difference of means^[7]^, BSSR in multi-armed clinical trials when the outcome variable is normally distributed^[8]^, BSSR with negative binomial counts in superiority and non-inferiority trials^[9]^, and BSSR with count data in multiple sclerosis^[10]^, etc.

However, significantly longer progression-free survival (PFS), overall survival (OS) and time to progression (TTP) are well known to be widely used in oncology studies as primary endpoints to evaluate the efficacy of treatment. Thus, the response variable of most oncology clinical trials is survival time. To the best of our knowledge, the use of BSSR in this context has rarely been reported. Here, we tried to develop an EM approach for BSSR with exponentially distributed outcomes. The performance and applicability of this procedure are described based on several simulation studies.

## Materials and methods

### Derived EM algorithm

The sample-size formula for an oncology clinical trial can be simplified if it is expressed as the number of deaths required rather than the number of patients. Suppose that a two-sided test will be performed with a significance level of *α*/2 and a power of 1-*β* for a hazard ratio Δ. Let *z*_1-*a*__/2_ and *z*_1-*β*_ be the 1-*α*/2 and 1-*β* percentiles of the normal distribution, respectively, and let *P*_*A*_ and *P*_*B*_ be the proportion of the patients randomized to treatments A and B, respectively. Then, the total number of deaths required is given by the following expression^[11]^:

[1] $${\left(Z_{1-\alpha /2}+Z_{1-\beta }\right)}^{2}/\left(P_{A}P_{B}\ln^{2}\Delta \right)$$

In general, only Δ in the above formula is unknown and needs to be estimated based on previous studies. However, if Δ is much larger or smaller than estimated, the sample size will need to be re-estimated without breaking the randomization codes. Gould and Shih proposed a EM algorithm-based procedure for blinded variance estimation for normally distributed endpoints^[6, 12]^; we extended this EM algorithm for Δ estimation to exponentially distributed endpoints without un-blinding the treatment group at the interim stage.

As the treatments are not identified, any interim observation, *x*_i_, *i* = 1,…,*n*, could be in either treatment group such that the treatment assignments are "missing at random". Let *t*_i_ denote the treatment group indicator, e.g., *t*_i_ = 1(0) indicates that sample i is in treatment group 1 (group 2); *t*_1,…_*t*_n_ are independent random variables with P(*t*_i_ = 1) = *q*. The density function of the exponential distribution is $f\left(t\right)=\lambda e^{-\lambda t}$, and l is the scale parameter. Given *t*_i_, *x*_i_ (*i* = 1,…,n) is distributed as follows, with density

(2) $$f\left(x_{i}|\tau_{i},\lambda_{1}{,\lambda }_{2}\right)=\lambda_{1}^{\tau_{i}}\lambda_{2}^{\left({1-\tau }_{i}\right)}exp\left[-\tau_{i}\lambda_{1}x_{i}-\left(1-\tau_{i}\right)\lambda_{2}x_{i}\right]$$

Therefore, the expression for the expected *t*_i_ given *x*_i_ is

(3) P(τi=1|xi)=P(τi=1)P(x=xi)P(τi=0)P(x=xi)+P(τi=1)P(x=xi)=θλ1exp(−λ1xi)θλ1exp(−λ1xi)+(1−θ)λ2exp(−λ2xi)=11+1−θθλ2λ1exp(λ1−λ2)xi

Then, the log-likelihood of the interim observations is

(4) $$L\left(\lambda_{1},\lambda_{2}|x_{i},\tau_{i}\right)=\overset{n}{\underset{i=1}{\sum }}\left\{\tau_{i}\ln\lambda_{1}+\left(1-\tau_{i}\right)\ln\lambda_{2}+\left[-\tau_{i}\lambda_{1}x_{i}-\left(1-\tau_{i}\right)\lambda_{2}x_{i}\right]\right\}$$

After taking the partial derivative of the above log-likelihood, the maximum likelihood estimates of *l*_*1*_/*l*_*2*_ are

(5) $$\Delta =\lambda_{1}/\lambda_{2}=\frac{\overset{n}{\underset{i=1}{\sum }}\tau_{i}}{\overset{n}{\underset{i=1}{\sum }}\tau_{i}x_{i}}/\frac{\overset{n}{\underset{i=1}{\sum }}\left({1-\tau }_{i}\right)}{\overset{n}{\underset{i=1}{\sum }}\left({1-\tau }_{i}\right)x_{i}}$$

The EM algorithm for estimating *λ*_1_/*λ*_2_ proceeds as follows. For example, if *θ* is assumed to be 0.5, then the "E" step consists of substituting the "initial" estimates of *λ*_1_ and *λ*_2_ into formula (3) to obtain provisional values for the expected value of *t*_i_. "M" consists of obtaining maximum likelihood estimates of *λ*_1_/*λ*_2_ according to (5) after replacing *t*_i_ in (5) with the provisional expectations. The "E" and "M" steps are repeated until the value of *λ*_1_/*λ*_2_ stabilizes.

### Simulations

We use simulations to evaluate performances of the proposed procedures. Specifically, various Δ estimators were compared for different scenarios. The censoring was not considered in the following simulations because the derived EM algorithm was used to re-estimate the number of events. For the EM algorithm, the recursive computation was continued until successive estimates of *l*_1_ and *l*_2_ differed by less than 0.001. Besides, we also designed a simulated clinical trial to investigate the power and illustrate BSSR procedure.

Firstly, the sample size requirement of the above EM algorithm was investigated. We considered the situation of equal samples for each distribution, population parameters were set as *l*_1_ = 0.1, 0.12, 0.15, 0.2, 0.25, 0.3 and *l*_2_ = 0.1, and several scenarios for the number of events per group differed from 5 to 800. The initial values were set to 1.0 and 0.8. In addition, the complete proportion of the total sample size in interim analysis is also an impact factor related to sample size. We defined Δ=0.66, 2.0, 3.0 to do investigation. It was set *α* = 0.05, power= 90%, we calculated the number of events using (1) for each scenario with 100% complete proportion, and the estimated values are listed in column "*n*" in the first line of ***Table 1***. The other "*n*" cells are calculated according to the defined complete proportion; for example, the 80% complete proportion means 80% of the total number of events are completed in interim stage, *n* is 103 for scenario *l*_1_ = 0.10 and *l*_2_ = 0.15 which is calculated by 129*80%.

**Tab.1:** EM re-estimation of **Δ ** with different completed proportions on 1,000 runs

**Completed proportion**	*****λ***_1_ = 0.10, ***λ***_2_ = 0.15 **		*****λ***_1_ = 0.2, ***λ***_2_ = 0.1 **		*****λ***_1_ = 0.3, ***λ***_2_ = 0.1 **	
	***n***	**Δ Mean**	**Δ SD**		***n***	**Δ Mean**	**Δ SD**		***n***	**Δ Mean**	**Δ SD**	
100%	129	0.6661	0.15923		45	2.0259	0.80409		18	3.0880	1.82815	
80%	103	0.6730	0.16825		36	2.0375	0.87898		14	3.1139	2.05301	
60%	77	0.6811	0.18731		27	2.0468	0.96885		11	3.2532	2.57707	
40%	52	0.6877	0.20607		18	2.1623	1.38557		7	3.5356	3.86211	
20%	26	0.7027	0.2418		9	2.4899	2.50656		4	5.0993	9.37600	

*λ*_1_ and *λ*_2_ are exponential distribution parameters, Δ is *λ*_1_/*λ*_2_.

Secondly, as the negligible impact of initialization on the EM estimation procedure, we investigated its effects by calculating the Δ estimates according to the above EM procedure for 1,000 repeated simulations with different values of the initialization constants of λ*_1_ and λ*_2_. We selected equal number of events per group (*n* = 50) for the internal pilot study.

Thirdly, the balanced design had been considered in most sample size estimation studies. Here, we investigated the impact of the sample allocation ratio on the above EM procedure. It was set with *α* = 0.05, power= 90%, and we calculated the number of events using (1) for each allocation ratio and the estimated values listed in column "*n*" of ***Table 2***. As some cells of "*n*" had the *n*_1_ or *n*_2_ values which were smaller than 20, considering the impact of the sample size, the number of events was expanded to at least 50 for one of the groups to obtain column "n*" in ***Table 2***. Δ was then re-estimated based on "*n*" and "n*", respectively.

**Tab.2:** EM re-estimation of D with different sample allocation ratio on 1,000 runs

**Parameters**	**Allocation Ratio**	***n***	**Δ Mean**	**Δ SD**		n*			**Δ Mean**	**Δ SD**	
*λ*_1_ = 0.2, *λ*_2_ = 0.1	3:1	*n*_1_ = 87,*n*_2_ = 29	1.9439	0.69021		*n*_1_ = 150,*n*_2_ = 50			1.9611	0.5554	
	2:1	*n*_1_ = 66,*n*_2_ = 33	1.9666	0.70763		*n*_1_ = 100,*n*_2_ = 50			1.9779	0.60863	
	1:1	*n*_1_ = 45, *n*_2_ = 45	2.0259	0.80409		*n*_1_ = 50, *n*_2_ = 50			2.0291	0.78344	
	1:2	*n*_1_ = 33, *n*_2_ = 66	2.1372	1.0208		*n*_1_ = 50, *n*_2_ = 100			2.0970	0.84075	
	1:3	*n*_1_ = 29, *n*_2_ = 87	2.2181	1.19095		*n*_1_ = 50, *n*_2_ = 150			2.1460	0.90141	
*λ*_1_ = 0.3 *λ*_2_ = 0.1	3:1	*n*_1_ = 36,*n*_2_ = 12	2.8674	1.34612		*n*_1_ = 150,*n*_2_ = 50			2.9581	0.68016	
	2:1	*n*_1_ = 28,*n*_2_ = 14	2.9109	1.38883		*n*_1_ = 100,*n*_2_ = 50			2.9712	0.77198	
	1:1	*n*_1_ = 18, *n*_2_ = 18	3.0880	1.82815		*n*_1_ = 50, *n*_2_ = 50			3.0060	1.03945	
	1:2	*n*_1_ = 14, *n*_2_ = 28	3.3216	2.46425		*n*_1_ = 50, *n*_2_ = 100			3.0630	1.10128	
	1:3	*n*_1_ = 12, *n*_2_ = 36	3.6878	3.96318		*n*_1_ = 50, *n*_2_ = 150			3.0660	1.16034	

*λ*_1_ and *λ*_2_ are exponential distribution parameters, Δ is *λ*_1_/*λ*_2_, n* is expanded sample size.

In a simulation study, we investigated the power and BSSR procedure with dummy randomized clinical trials in which the survival data of the two treatment groups were compared. We obtained the independent and identically distributed time-to-event observations from exponential distributions and the censored data from uniform distribution.

In our design, an initial sample size (*n*_0_) was carried out based on assumed parameters. Although this sample constituted the final sample size for the fixed design, the sample size could be adjusted using BSSR based on half of the initially sample size, i.e., 50% information time. The following parameter values were considered: assumed median survival time T = 10 and 5 for the treatment group and the control group, respectively; therefore, the scale parameters *λ* of the exponential distributions were calculated to be 0.07 and 0.14 based on the formula *λ* = log (2)/T. Two different scenarios were considered for the true median survival time, T' = 10, 5 and T' = 12, 5. Furthermore, the design was balanced and the significance level and target power were the usual *α*= 0.05 (two-sided) and 1-*β* = 0.9.

The number of failures for the fixed design was estimated to be 45^[13– 14]^ per group based on the assumed parameters, and the internal pilot study included 23 failures per group for an information time of 50%. The assumed censor rates were 0, 20%, and 40%, and then the corresponding event rates were 100%, 80%, and 60%, respectively. Accordingly, these values resulted in fixed sample sizes (*n*_0_) of 45, 56, and 75, respectively, based on sample size= number of events/(event rate). The true parameters were used to generate simulated data with a fixed sample size (*n*_0_). According to EM algorithm, the blinded re-estimated sample size (*n*_1_) was based on the 23 (the half of 45) events from an assumed internal pilot study which is defined as 50% information time of the integrity trial^[15]^. Subsequently, 1,000 trails were simulated from each scenario.

## Results

### Sample size requirement

**Fig.1:**
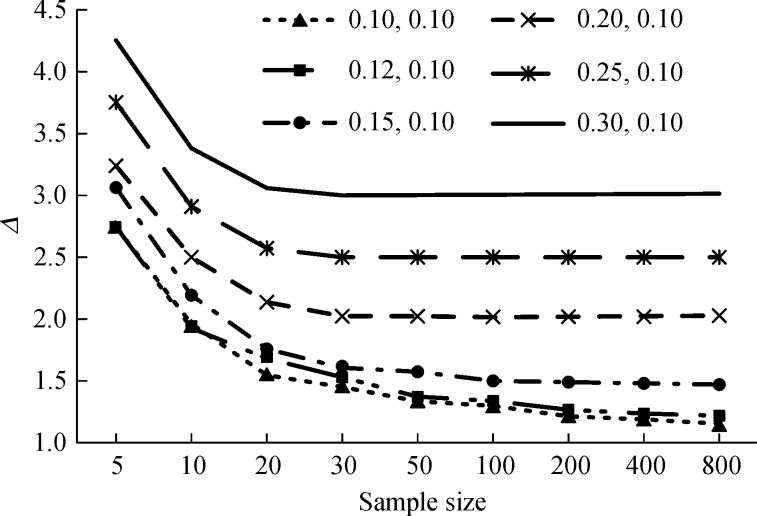
EM re-estimation of Δ with different sample sizes

***Fig. 1*** shows the EM re-estimation of Δ for different number of events; 1,000 simulation replications were performed for each situation. The estimation results stabilized as the number of events increased. All results were overestimated when the number of events per group was less than 30. The smaller Δ, the higher sample size was required. More than 100 sample sizes was needed when Δ was 1.5. For Δ = 1.0, there was about 16.05% overestimation even if sample size reached 800, this result was similar with Xie's research (13.54%)^[16]^.

***Table 1*** also shows that the estimates were impacted by event numbers (sample size). For *λ*_1_ = 0.10 and *λ*_2_ = 0.15, the estimates were all acceptable even if the completed proportion was 20%, which nearly satisfied the sample size requirement of this EM algorithm. On the contrary, the estimates significantly deviated from the real values for *λ*_1_ = 0.3 and *λ*_2_ = 0.1 if the completed proportion was less than 80% due to the number of events was insufficient for the EM algorithm.

### Initial values

The simulation results are presented in ***Table 3***. Specifically, the estimates obtained from the EM procedure depended on the initialization. It shows that the Δ estimates exceeded 1 if λ*_1_>λ*_2_ and were less than 1 if λ*_2_<λ*_1_. Fortunately, the estimated Δ values were very close to the true values when the initial values satisfied λ*_1_>λ*_2_ with the true values *λ*_1_>*λ*_2_, and λ*_1_<λ*_2_ with *λ*_1_<*λ*_2_. The estimated number of the required events using (1) was equal for both Δ = *λ*_1_/*λ*_2_ and Δ = *λ*_2_/*λ*_1_. Therefore, the choice of λ*_1_>λ*_2_ or λ*_1_<λ*_2_ did not affect the re-estimation of the number of events. ***Table 3*** also shows the overestimation results for Δ = 1 (*λ*_1_ = 0.2, *λ*_2_ = 0.2), and the choice of initial values did not affect these overestimated results.

**Tab.3:** EM re-estimation of Δ with different initial values on 1,000 runs

**Initial values**	***λ*_1_ = 0.2, *λ*_2_ = 0.1 **		***λ*_1_ = 0.1, *λ*_2_ = 0.2 **		***λ*_1_ = 0.2, *λ*_2_ = 0.2 **		***λ*_1_ = 0.3 *λ*_2_ = 0.1 **		***λ*_1_ = 0.1 *λ*_2_ = 0.3 **
	**Δ Mean**	**Δ SD**		**Δ Mean**	**Δ SD**		**Δ Mean**	**Δ SD**		**Δ Mean**	**Δ SD**		**Δ Mean**	**Δ SD**
λ*_1_ = 1.0, λ*_2_ = 0.2	2.0299	0.78558		2.0118	0.75331		1.3324	0.51644		3.0169	1.05123		2.9939	0.99592
λ*_1_ = 0.8, λ*_2_ = 0.2	2.0301	0.78542		2.0121	0.75315		1.3197	0.51084		3.0169	1.05125		2.9938	0.99575
λ*_1_ = 0.4, λ*_2_ = 0.2	2.0283	0.78143		2.0109	0.75004		1.3494	0.54675		3.0020	1.03824		2.9794	0.98333
λ*_1_ = 0.2, λ*_2_ = 0.4	0.5694	0.21396		0.5704	0.21015		0.8178	0.22699		0.3814	0.15811		0.3810	0.15433
λ*_1_ = 0.2, λ*_2_ = 0.8	0.5692	0.21413		0.5703	0.21036		0.8093	0.22995		0.3803	0.15873		0.3799	0.15491
λ*_1_ = 0.2, λ*_2_ = 1.0	0.5693	0.21428		0.5705	0.21049		0.8260	0.22439		0.3803	0.15869		0.3799	0.15504

*λ*_1_ and *λ*_2_ are exponential distribution parameters, Δ is *λ*_1_/*λ*_2_, *λ*_1_* and *λ*_2_* are initial values.

### Sample allocation ratio

***Table 2*** results indicate that the EM estimates of Δ were impacted by the sample allocation ratio. The balanced design produced the best estimates. The unbalanced designed estimates, including 2:1, 1:2, 3:1 and 1:3, were slightly larger or smaller, and the sample size difference between the two groups directly correlated with the estimated values. More unbalance would get more biased estimation although the number of events increased to at least 50 for one group in "n*" column.

### Example (simulated)

***Table 4*** shows the statistical power tested by the log-rank test and exponential regression for fixed design and sample sizes re-estimated adjusted design. For the true median survival times of T' = 12 and 5, the original sample sizes were 45 for a 100% event rate, 56 for an 80% event rate and 75 for a 60% event rate, as shown in column "*n*_0_". Thus, these sample sizes were overfull to obtain 90% power for the log-rank test or exponential regression. Therefore, the power of the log-rank test and exponential regression exceeded 90% significantly. After a Δ re-estimation using the above EM algorithm, the number of events was re-calculated. Based on the pre-defined event rates (60%, 80% and 100%), the corresponding sample sizes were re-calculated and were shown in column "*n*_1_". From the output, the re-estimated sample sizes "*n*_1_" were near the actual requirement, the power for the adjusted design was closer to 90% for a 100% event rate.

**Tab.4:** Simulated power for fixed and adjusted designs

**scenario**	**True parameters**	**Event rate**	***n***_**0**_	**Fixed design Power (%)**		**Re-estimated**		**Adjusted design Power (%)**
	***λ***_**1**_			***λ***_**2**_	***T*****'**_**1**_	***T*****'**_**2**_	***Δ'***	**LogRank**	**Exponential Regression**		**Δ**	***n***_**1**_		**LogRank**	**Exponential Regression**
1	0.07	0.14	10	5	0.5	100%	45	89.7	89.3		0.512	46		90.2	91.6
2						80%	56	92.1	92.9		0.510	58		92.5	92.1
3						60%	75	94.8	94.0		0.510	77		95.5	94.9
4	0.06	0.14	12	5	0.42	100%	45	98.0	98.2		0.439	31		91.1	93.2
5						80%	56	99.3	99.5		0.430	36		93.4	94.0
6						60%	75	99.6	99.5		0.448	55		97.2	98.9

***λ***_1_ and ***λ***_2_ are exponential distribution parameters, *T*'_1_ and *T*'_2_ are true median survival times, Δ' is true hazard ratio, Δ is re-estimated hazard ratio, *n*_0_ is original sample size, *n*_1_ is re-estimated sample size.

**Fig.2:**
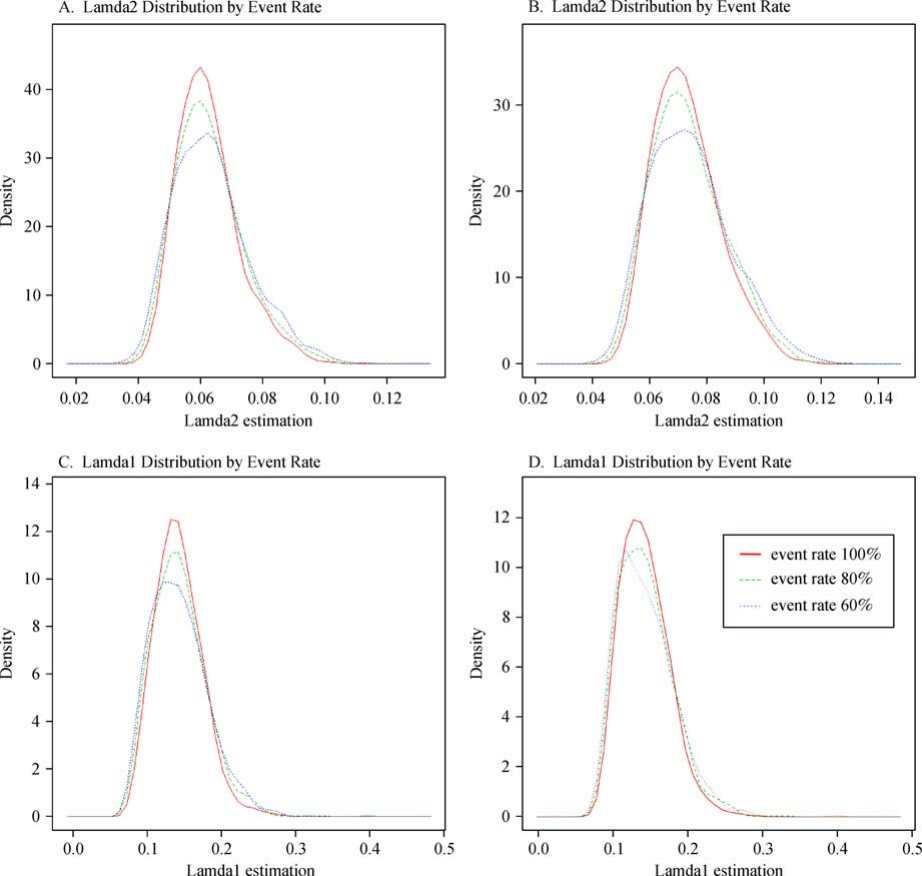
Kernel Density of ***λ***_1_ and ***λ***_2_ estimation.

For true median survival times of T' = 10 and 5, "*n*_0_" was the correct required sample size, and the values of re-estimated sample sizes "*n*_1_" were similar to the "*n*_0_" values; the power for both fixed design and adjusted design was close to 90% for a 100% event rate. The variation details of the simulated EM estimated values of ***λ***_1_ and ***λ***_2_ for each scenario are presented in ***Fig. 2***.

Notably, the power for both the log-rank test and exponential regression inversely correlated with the event rate in each scenario because the sample size calculation was based on the event numbers/(event rate). Lower event rates required larger samples, which increased the power of the test.

## Discussion

The topic of sample size reassessment during an ongoing trial has become very popular in recent years. The inflation of the type I error rate and the loss of power has long been an intractable problem for sample size adjustment in an internal pilot study with adaptive design. In the ICH E9 guidelines, it is reflected by the following requirement for planned sample size adjustment: "The step taken to preserve blindness and consequences, if any, for the type I error and the width of confidence intervals should be explained". The calculation of the actual type I error rate for the blinded case was previously derived for the *t-*test situation^[17]^, and the results showed that the nominal inflation in the type I error rate did not significantly differ for the blinded sample size recalculation in the unrestricted design. Over the last decade, a number of studies had addressed the problem of BSSR for an ongoing clinical trial. These documents noted that the assumptions of sample size calculation should be reassessed with the blinded data and that the effect on the type I error rate can be controlled^[18]^. To our knowledge, this work is restricted to the normally or binomially distributed data.

Cancer is currently one of the major diseases affecting human health, and anti-cancer drug becomes the focus of research in the pharmaceutical industry. Survival time-related outcomes such as PFS and OS are generally used as primary endpoints in oncology studies to assess efficacy. Thus, oncology trials usually require long follow-up periods and include a planned interim analysis, and sample size re-estimation is also essential. In this paper, we tried to extend BSSR method for exponentially distributed survival data based on EM algorithm.

The blinded data of an ongoing trial is from a mixture of two populations; one for the treatment group and the other for the control group. EM method described here is applied to estimate model parameters of the mixture distributions and therefore assess the hazard ratio. The derived EM estimation only can be used to re-estimate the number of events for oncology trials, and the censoring is ignored. Normally, it is acceptable in the clinical trial because the censor rate is always unknown and only assumed in the design stage. With the re-estimated number of events in the interim stage, the total censor rate of the two treatments which is available without breaking blind, could be used to do sample size re-estimation, because sample size= the re-estimated number of events/(1-interim stage censor rate). On the other hand, only exponential distribution has been considered in our research, and Weibull distribution needs to be investigated in the future since it is more widely applied in survival analysis.

Our studies show that estimates from the EM method are highly variable, which coincides with the literature on interim analysis of treatment effects with blinded data and a normally distributed endpoint. Besides, the relatively small hazard ratios (Δ<1.2) are overestimated according to this EM method.

Specifically, for the hazard ratios (Δ≥1.2), the EM estimation shown here is subject to a sample size requirement and the stability of the estimation results directly correlated with the number of events. The initial values for the parameters directly affect the convergence of the algorithm and the estimated results. The simulation shows that the initial values should be carefully selected, and the calculation of the optimum initial values requires further research. Moreover, the EM estimation described here is more suitable for a balanced design.

Due to the variation and overestimation of smaller hazard ratios, no reliable inference can be made on sample size re-estimation in our studies. The results from this paper provide useful information to steer the practitioners in this field from repeating the same endeavor. This should be of some relief to health authorities.
